# 3D printing of twisting and rotational bistable structures with tuning elements

**DOI:** 10.1038/s41598-018-36936-6

**Published:** 2019-01-23

**Authors:** Hoon Yeub Jeong, Soo-Chan An, In Cheol Seo, Eunseo Lee, Sangho Ha, Namhun Kim, Young Chul Jun

**Affiliations:** 1 School of Materials Science and Engineering, Ulsan National Institute of Science and Technology (UNIST), Ulsan, 44919 Republic of Korea; 2 School of Mechanical, Aerospace and Nuclear Engineering, UNIST, Ulsan, 44919 Republic of Korea

## Abstract

Three-dimensional (3D) printing is ideal for the fabrication of various customized 3D components with fine details and material-design complexities. However, most components fabricated so far have been static structures with fixed shapes and functions. Here we introduce bistability to 3D printing to realize highly-controlled, reconfigurable structures. Particularly, we demonstrate 3D printing of twisting and rotational bistable structures. To this end, we have introduced special joints to construct twisting and rotational structures without post-assembly. Bistability produces a well-defined energy diagram, which is important for precise motion control and reconfigurable structures. Therefore, these bistable structures can be useful for simplified motion control in actuators or for mechanical switches. Moreover, we demonstrate tunable bistable components exploiting shape memory polymers. We can readjust the bistability-energy diagram (barrier height, slope, displacement, symmetry) after printing and achieve tunable bistability. This tunability can significantly increase the use of bistable structures in various 3D-printed components.

## Introduction

3-dimensional (3D) printing, also called additive manufacturing, is a new paradigm in customized manufacturing^1–3^. An arbitrary 3D object with complexities in shape and material can be built layer-by-layer from the bottom up. However, most components fabricated by 3D printing so far have been static structures with fixed shapes and monotonic functions. One possible route for precisely controlled, reconfigurable structures is to use structural multistability, allowing multiple stable shapes. In such structures, reversible switching between stable configurations is possible under proper mechanical actions. A bistable structure is one that supports two stable states. Such bistability has been studied in strained bilayers^4–6^, origami-based structures^7–9^, and compliant mechanisms^10–13^. Bistability exists in nature too^14,15^. Reconfigurable, adaptive structures can be widely used for diverse applications. Bistable structures do not need additional energy to maintain their stable states. Therefore, they can work as mechanical switches. They can also simplify actuation and motion control^16–18^, and be used to rapidly induce large-magnitude movements in a pre-designed way. This can reduce the number of parts, weight, and assembly time of actuators while increasing precision and reliability.

In this work, we propose a new approach to tunable bistable components using shape memory polymers (SMPs). We first present 3D-printed bistable structures that allow twisting and rotational reconfiguration. Our designs are based on compliant mechanisms but, unlike conventional monolithic structures without joints, we introduce special joints that allow 3D printing of whole components without post-assembly. These joints are also important for the tuning of bistability. Then, by employing SMP elements, we demonstrate tunable bistable components. SMPs are smart materials that can memorize a permanent shape^19,20^. In fact, they can have multiple, arbitrary temporary shapes; SMPs soften above the glass transition temperature (T_g_) and allow reshaping. A new shape can be fixed by cooling back to room temperature, where SMPs exhibit significant stiffness. We apply this unique property of SMPs to bistable structures to enable tunable components. Using tunable SMP elements embedded in twisting and rotational components, we can adjust twisting or rotational angles and control the overall shape of the bistability-energy diagram. We can readjust the bistability-energy diagram (barrier height, slope, displacement, symmetry) after printing and achieve tunable bistability. This can significantly increase the use of bistable structures in various 3D-printed components. These tunable bistable components can be useful for simplified motion control in actuators or for mechanical switches.

## Results and Discussion

### 3D printing of bistable structures without post-assembly

**Figure 1:**
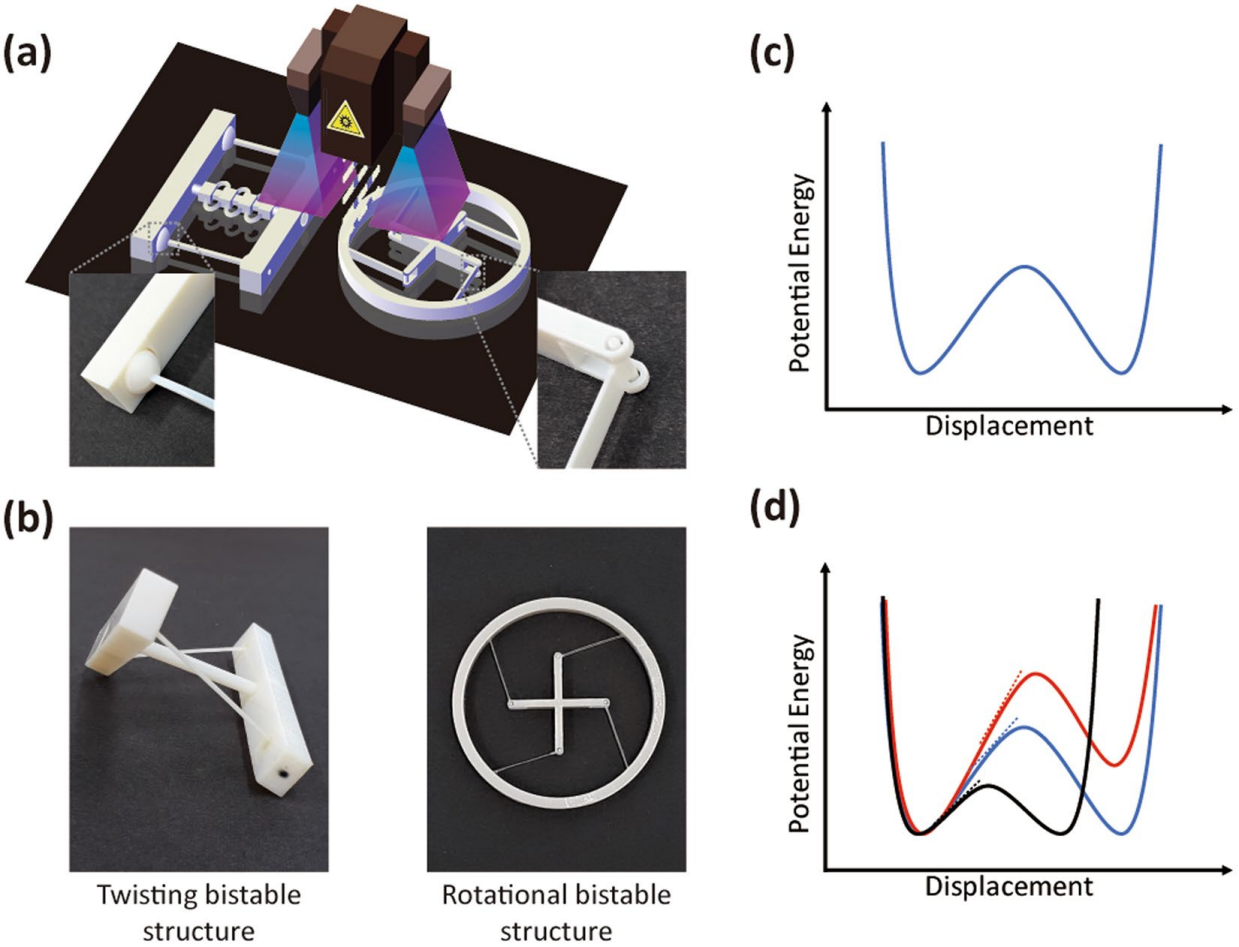
3D printing of reconfigurable, bistable structures without post-assembly. (**a**) Schematic for 3D printing of twisting and rotational bistable components. Inset: pictures of the ball joint for twisting components (left) and the pin joint for rotational components (right). These joints enable 3D printing of the whole bistable components without post-assembly. (**b**) Pictures of twisting and rotational bistable structures. (**c**) Schematic for a bistability energy diagram (i.e. deformation energy diagram). Two stable states are separated by a well-defined energy barrier. To induce transformation into the other state, we should apply the force large enough to overcome this barrier. (**d**) By adjusting bistable structures and material parameters, we can control the barrier height (i.e. threshold energy for a shape change), the slope (i.e. force), the amount of displacement to induce a shape change, etc. Therefore, it is a key issue to understand and tailor the shape of bistability energy diagrams for highly controlled reconfiguration.

Figure 1(a) presents a schematic for the 3D printing of bistable components. The polyJet process, by which photopolymer-ink droplets are jetted and cured with ultraviolet light, was used in a Stratasys multi-material 3D printer (J750). The polyJet process allows the creation of fine features with a resolution of about 50 μm in the plane and 15 μm in thickness. Geometric design was first created using CAD software and then imported into the Stratasys printer. Twisting and rotational bistable components were fabricated using consolidated design without post-assembly. To this end, special joints were used to allow 3D printing of whole components; ball joints were employed in twisting components, pin joints in rotational ones (insets in Fig. 1(a)). These joints also allowed us to design tunable bistable components. The joint structures were implemented by applying supporter materials to separate and move two consolidated parts. Such materials can be dissolved in water or removed easily after printing, e.g., using water jetting, and thus they can enable construction of a complex 3D structure of consolidated parts without an additional assembly process.

There have been recent works on 3D-printed local instabilities that could be useful for deployment and energy absorption^21–24^. However, these works were mainly limited to simpler translational motion. Here, we consider global bistability allowing twisting and rotational movements in 3D-printed structural elements (Fig. 1(b)). To realize rotational bistability, the exact shape of the deformed beam was calculated by solving the nonlinear equations in beam theory. We used commercial materials for our printing. The rigid supporting bodies (rectangular bar and central cylinder for twisting structures, outer ring and inner cross for rotational structures) are made of digital ABS or VeroWhite.

### Elastic-potential energy diagram for bistable components

Bistable structures have two stable states that are separated by an energy barrier, as shown in Fig. 1(c). These stable states are the local minima of the elastic-potential-energy diagram. Switching between two stable configurations (states A and B) can be done reversibly many times through proper mechanical actions with lateral or rotational forces. The slope in the potential-energy diagram indicates the force applied at a given displacement. To overcome the energy barrier and induce transformation into the other shape, we should apply enough energy to overcome this barrier. Once we pass the hill of the barrier, the structure will be deformed into another stable, lower-energy state automatically without additional energy. This can be, for example, used as mechanical switches or actuators for simplified and precise motion controls (e.g., in robots) without the use of complicated position- and force-control systems. A bistable structure remains stable over time without energy consumption because it is in a stable-energy position. Small disturbances do not change the stable position; therefore, an open-loop motion control system is adequate for accurate motion control. Figure 1(d) explains this idea further. The shape of the bistability-energy diagram is determined by structural design, as well as material properties. By adjusting bistable structures and material parameters, we can control the barrier height (i.e., the threshold energy for a shape change), the slope of the barrier (i.e., the force required for a shape change) and the amount of initial displacement for a shape change. The symmetry in the energy diagram also determines whether the threshold energies are equal in each direction (A → B or B → A). If the energy diagram is asymmetric, one direction has a smaller energy barrier than the other, meaning that the transition in one direction is easier than that in the other. Therefore, it is a key issue to understand and tailor the shape of the bistability-energy diagram for highly controlled reconfiguration.

### Twisting bistable components

**Figure 2:**
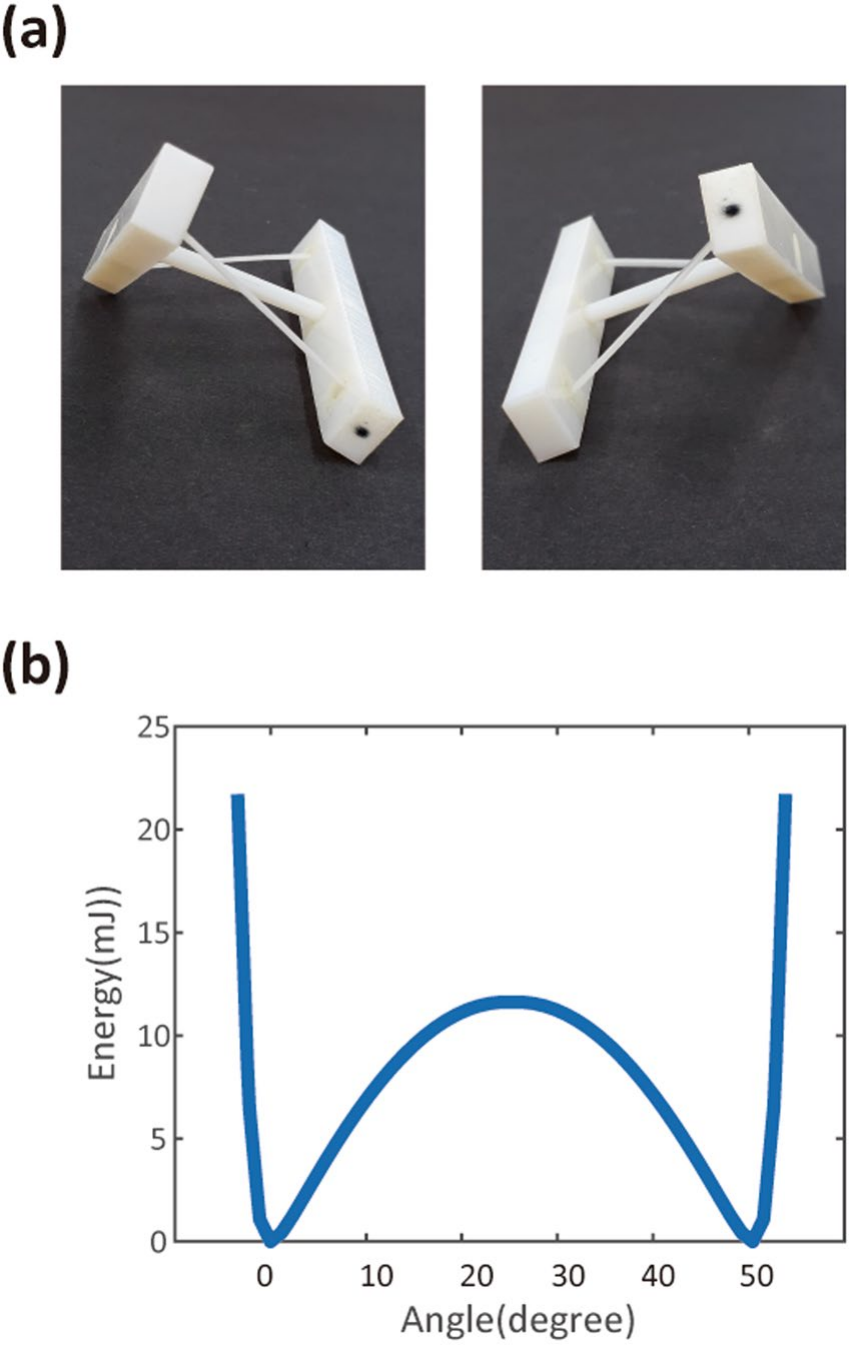
Twisting bistable components. (**a**) Picture of 3D-printed, twisting bistable components. The right rectangular bar is indicated by a small black dot for eye tracing. (**b**) Elastic-potential energy diagram from FE analysis.

We first consider twisting bistable structures (Fig. 2). Two rectangular bars in the twisting structure are connected by two thin beams (beam thickness: 0.8 mm) and a central cylindrical rod (Fig. 2(a)). The two beams are ball-jointed, allowing them to rotate freely. With enough energy, the structure can be twisted into the other stable configuration. The energy required to overcome the barrier can be modified using other materials, or by changing the thickness of the beam. Supplementary Video 1 and 2 show the flipping of the 3D-printed component between these two stable states (Supplementary Figure S1). Flipping between them can be repeated. In particular, once we pass the hill of the barrier, the structure transforms rapidly into the other stable state without further energy supply, also called snap-through instability^22,25,26^. We performed the finite element (FE) analysis and obtained the elastic-potential-energy diagram for this twisting structure (Fig. 2(b)). For simplicity, joints were assumed to be frictionless, and the parts undergoing translational and rotational motion were regarded as rigid bodies. We obtained two clear-energy minima separated by an energy barrier. However, we note that the shape of each energy-minimum pocket is not exactly symmetric; the potential increases abruptly on the leftmost and rightmost sides of the energy diagram. This is because the beams cannot be easily extended further once they are fully straightened. The moduli of all printing materials used in the current work were measured using DMA (see Methods).

### Rotational bistable components

**Figure 3:**
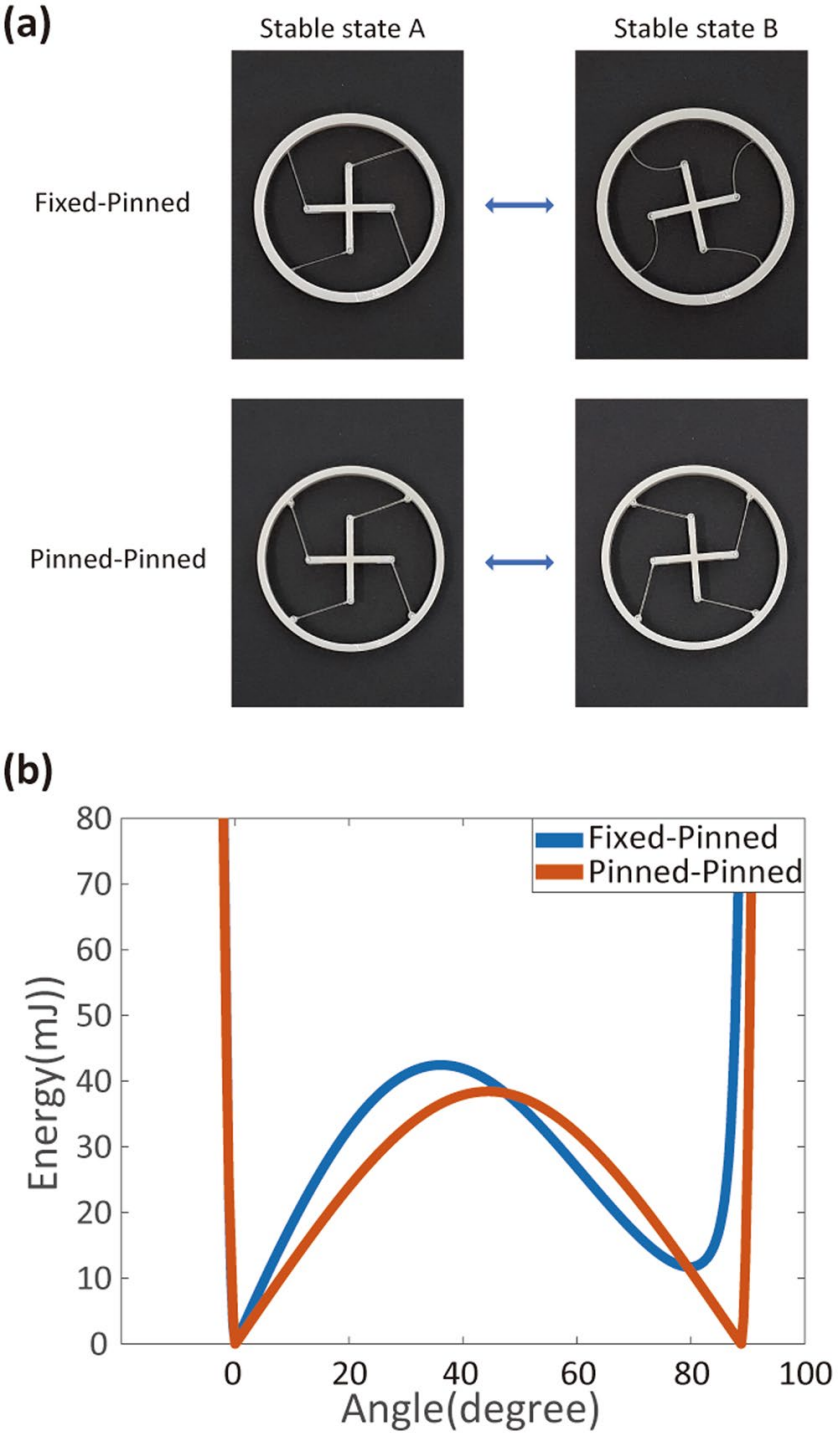
Rotational bistable components. (**a**) Picture of 3D-printed, rotational bistable components with fixed-pinned beams (upper) and pinned-pinned beams (lower) (beam thickness: 0.5 mm). The rotation angles between two stable states are measured to be 79° and 87° for fixed-pinned beams and pinned-pinned beams, respectively. (**b**) Elastic potential energy diagram for rotational bistable components.

Figure 3 shows 3D-printed rotational bistable components. The inner cross is connected to the outer ring by four beams. The inner part can be inter-rotated into two stable states, while the outer ring is held on. To adjust the rotational angles and the shape of the energy diagrams, we used different boundary conditions at the connections between the beams and the inner and outer parts. The 3D printing of these rotational structures is more straightforward than twisting the structures, because the whole structure is on the same plane and is thus better for layer-by-layer printing. In order to implement the second stable state, it is necessary to know the exact shape of the deformed beam. This shape was obtained by solving the nonlinear equation from the beam theory (see Supplementary Figure S2).

To realize rotational bistability, we used either fixed (i.e., clamped) or pinned (i.e., freely rotating) boundaries. In Fig. 3, we show rotational components with fixed–pinned beams (upper) and pinned–pinned beams (lower). For each case, we computed the energy diagrams again. The fixed–pinned structure has an asymmetric energy diagram (blue curve in Fig. 3(b)), because two stable states A and B have different shapes. The state A is an as-printed structure without deformation. However, the second stable state (B) is still under stress; therefore, the local minimum at B has a higher energy than A. On the other hand, the pinned–pinned beam has a symmetric energy diagram (red curve in Fig. 3(b)), because the two stable states now have identical shapes due to the freely rotating, pinned boundaries. We can think of another possible case too: that of fixed–fixed beams (Supplementary Figure S3). This configuration also supports bistability, but the rotational angle is very small and the energy barrier is much lower than the others. Thus, we do not consider it here.

### Tunable bistable structures with SMP elements

Now, we introduce SMP-based tuning elements into our components and demonstrate tunable bistability. SMPs can possess various temporary shapes; a temporary shape is fixed during a glass-transition or crystallization process while retaining strains internally. By heating above a transition temperature again (the glass-transition temperature, T_g_, in our case), they soften and the internal strains are released.

**Figure 4:**
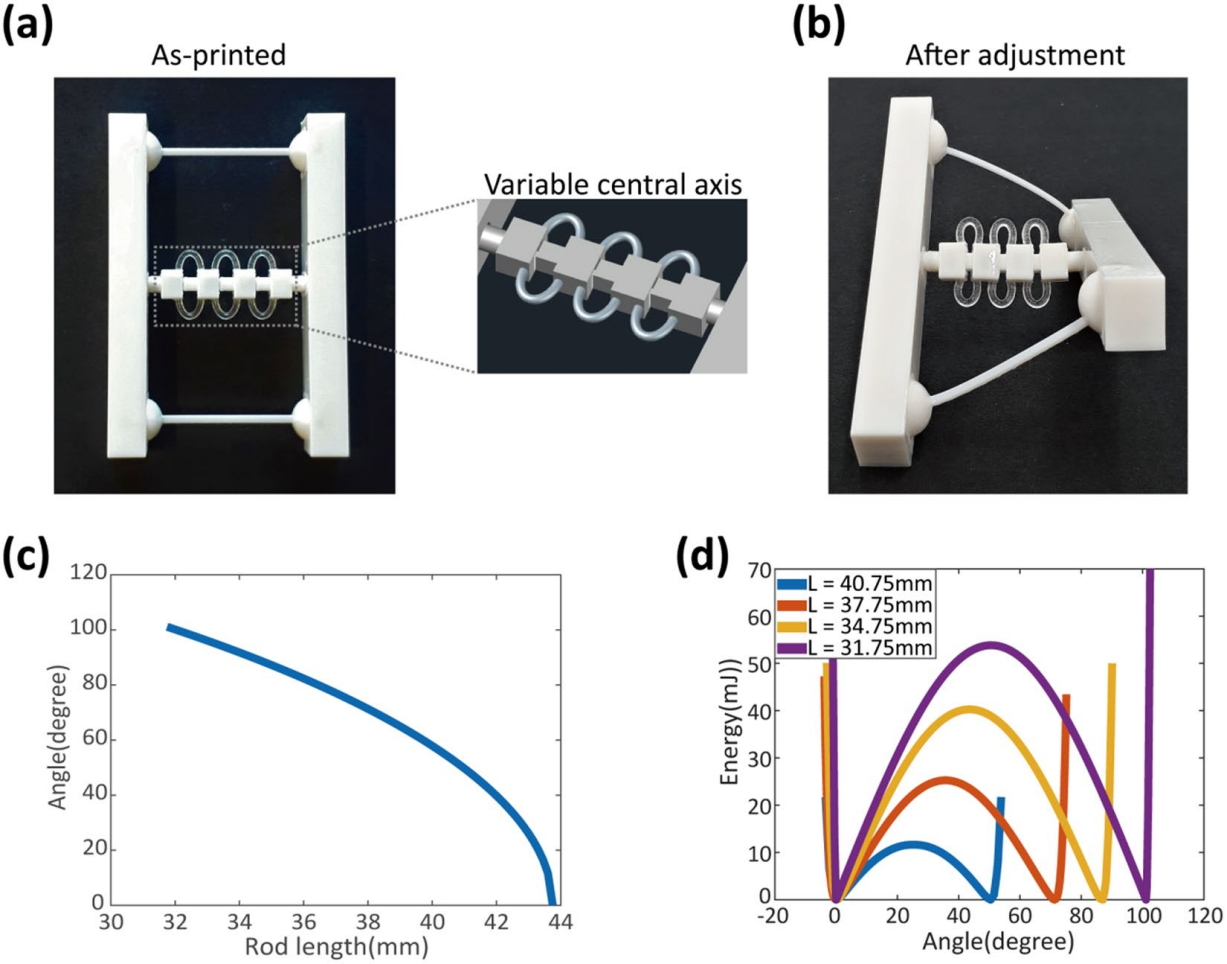
Tunable bistability in twisting structures with shape memory polymer (SMP) tuning elements. Pictures of (**a**) the as-printed, flat structure and (**b**) the twisting structure after adjustment [Inset in (**a**): CAD image of the tuning element]. The central cylindrical rod is printed in a tunable geometry using SMP half rings. The SMP softens above the glass transition temperature (T_g_), and we can readjust the length of the central rod and then fix the shape by cooling it back to room temperature, where the SMP exhibits significant stiffness. (**c**) The length of the central cylindrical rod determines the twisting angle. (**d**) The bistability energy diagram changes too with the central rod length (L). The original length of both twisting beams and the central rod is 43.75 mm. The length of the central rod can be reduced to 31.75 mm. As the rod length decreases, two energy minima get more separated (i.e. resulting in a larger twisting angle) and the energy barrier gets higher, which means we need to apply more force to overcome the barrier. The overall energy diagram remains symmetric.

A twisting bistable structure is again 3D-printed; now, however, the central cylindrical rod is printed in a tunable geometry using SMP elements, as shown in Fig. 4(a). This length-adjustable central rod consists of segmented parts and connecting rings. The segmented parts can be plugged together while the connecting rings are bent further. The half rings in tuning elements are made of a digital SMP material (RGD8630-DM). The SMP softens above T_g_, and we can reduce the length of the central rod by bending the SMP connecting rings and then fixing the shape by cooling it back to room temperature, where the SMP exhibits significant stiffness.

Supplementary Video 3 shows the tuning and flipping of this bistable structure and confirms twisting bistability. This temporary configuration is fixed until it is again exposed to heat. By adjusting the central rod’s length, we can tune the twisting angle (Fig. 4(b)). Initially, the whole structure is printed on the same plane. If the length of the beams and cylindrical rod are exactly the same, the structure cannot be twisted and all parts will be laid on the same plane – i.e., a flat structure, where bistability does not exist. Note that this is different from the previous one (Fig. 1(b)), in which the twisting component was directly printed out-of-plane using supporter materials, which were then removed after printing. Therefore, 3D printing of flat bistable components has the advantages of being more straightforward and saving both time and printing material.

**Figure 5:**
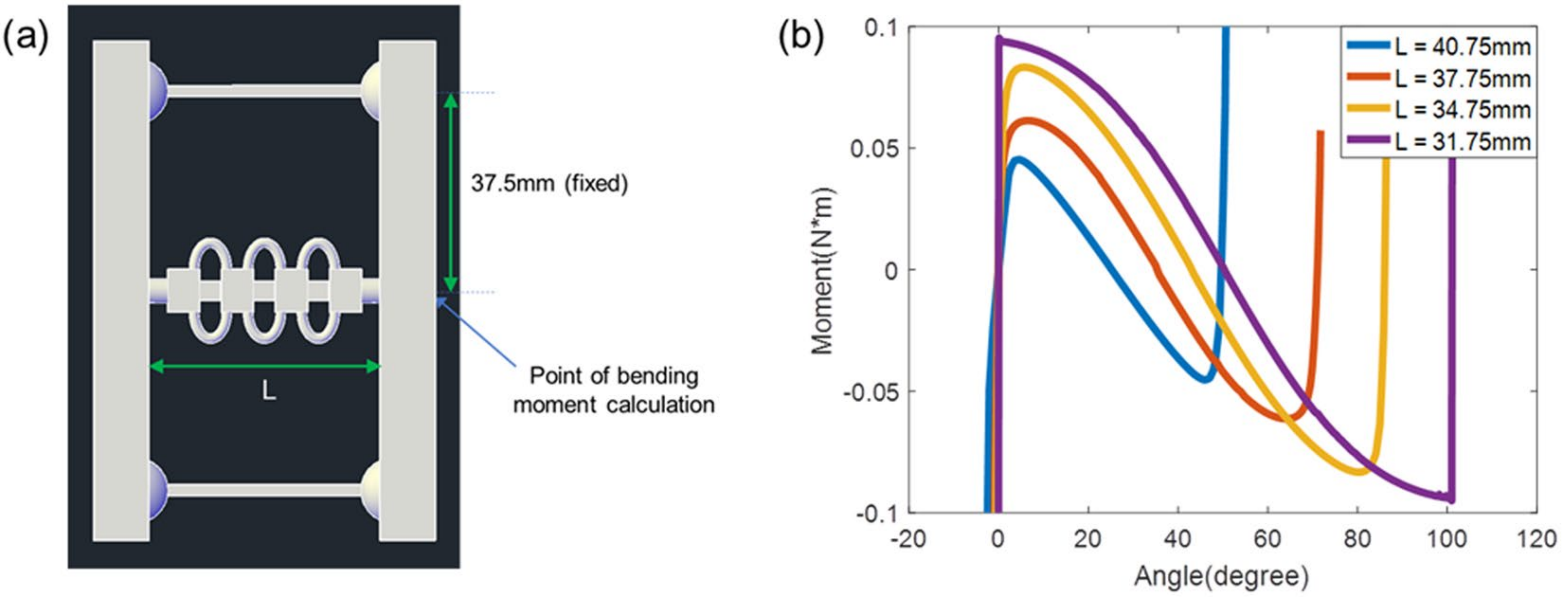
(**a**) Geometry of the tunable twisting structure. (**b**) Bending moment vs twisting angle. From simulations, we also obtained the bending moment as a function of twisting angle. The point of bending moment calculation is shown in (**a**). The central rod length L is gradually varied, while the center-to-ball joint distance is fixed as 37.5 mm. Note that the angle here refers to the twisting angle of our ‘global’ bistable structure.

The length of this central cylindrical rod determines the twisting angle (Fig. 4(c)). This twisting angle can be obtained as a function of the central rod length as explained in Supplementary Fig. S4. As the central-rod length decreases, the total twisting angle also gradually increases. Figure 4(d) also shows how the bistability-energy diagram changes with the central-rod length. When the rod length decreases, two energy minima become further separated (i.e., resulting in a larger twisting angle) and the energy barrier grows higher, meaning more energy is required to overcome this barrier. However, the overall energy diagram remains symmetric. From simulations, we also obtained the bending moment as a function of twisting angle (Fig. 5).

### Tunable rotational bistable components

**Figure 6:**
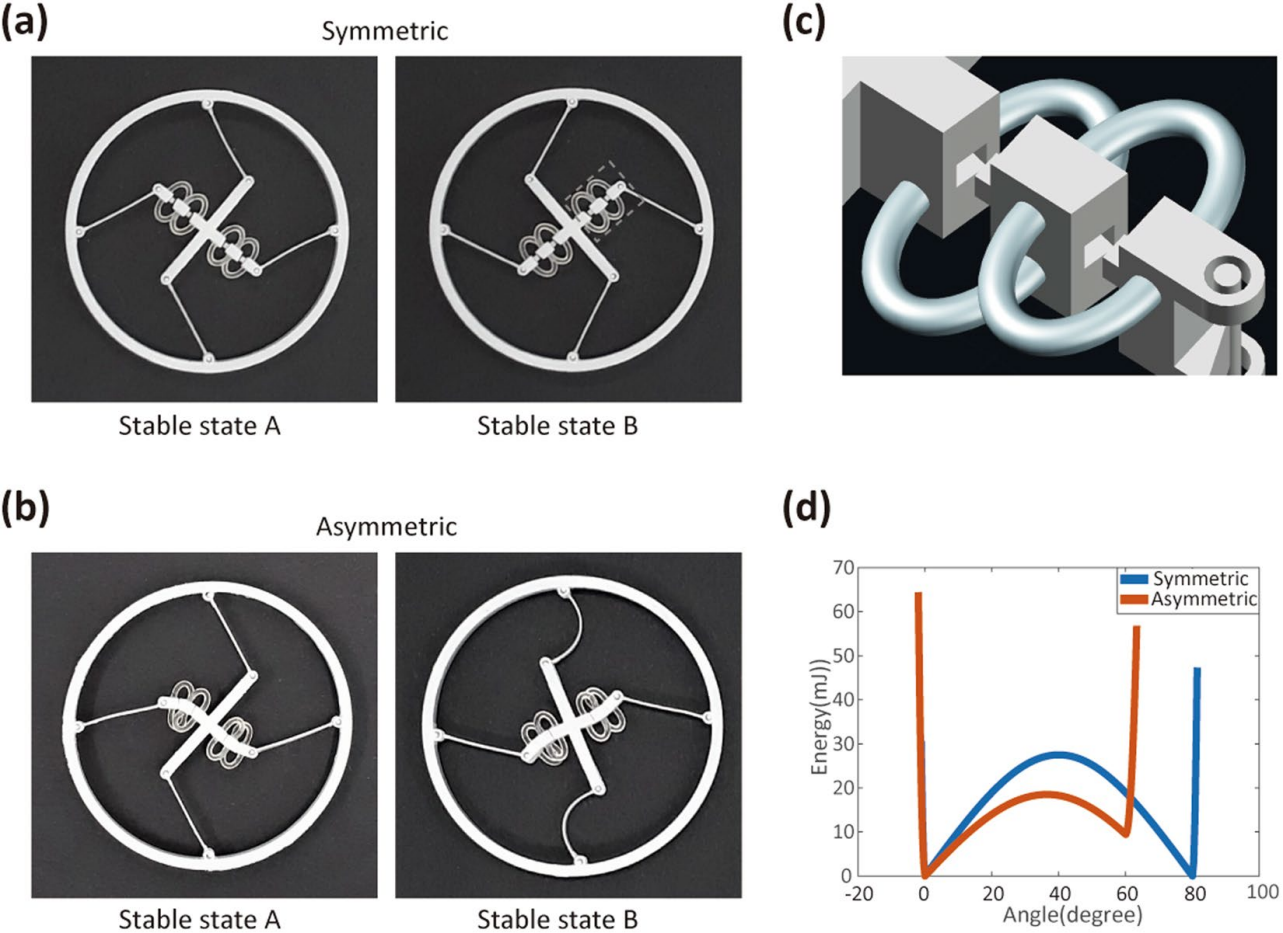
Tunable bistability in rotational structures and symmetry control in the energy diagram. Pictures of bistable structures (**a**) before adjustment (i.e., as-printed) and (**b**) after adjustment. The rotational structure with pinned-pinned beams is created including tunable SMP elements in the one arm only. (**c**) CAD image of the tuning element [corresponding to the dotted, gray square in (**a**)]. (**d**) The energy diagram is originally symmetric (blue curve), but with the reduced arm length, it becomes asymmetric (red curve). We solved equations to determine the required arm design.

Now we demonstrate tunable bistability in rotational components. As shown in Fig. 6(a,b), we prepared another rotational structure that includes tunable SMP elements in a one arm only. This has a pinned–pinned boundary, so the energy diagram was originally symmetric. By reducing the arm length, we can now adjust the symmetry of the bistability-energy diagram. To this end, we designed the tuning arm to be slightly curved when reduced. As shown in the CAD image in Fig. 6(c), the tuning arm is segmented into different parts and the end of each joining part is slanted. Thus, when the arm is plugged together by bending the SMP connecting rings, it curves naturally. By reducing the length of one arm in this way, we can obtain another set of two stable configurations (Fig. 6(b)), and thus achieve an asymmetric energy diagram. In Fig. 4, the energy diagrams remained symmetric while we adjusted the tuning angles and barrier heights. This time, however, we adjusted the symmetry of the energy diagram from symmetric to asymmetric; the barrier in one direction is lower than that in the other. The use of SMPs in this manner enhances the tunability and applicability of bistable structures. To design a tunable rotational bistable structure, we need to solve equations to determine the required arm length in the tuning element. This procedure is explained in Supplementary Fig. S5.

Our bistable structures may be mechanically tested using torsional strain sensors with a proper rotational shaft, which can be 3D-printed together. However, in conventional instruments, the force range is usually too high to be used for 3D-printed components. So, it would require a customized setup with a well-aligned strain gauge to precisely test the rotational or twisting structures.

## Conclusions

In conclusion, we described new ideas and concepts for 3D-printed bistable components, which are important for precise motion control and reconfigurable structures. Bistability produces a well-defined energy diagram, which dictates the movement in a pre-determined way. Therefore, these bistable structures can be useful for mechanical switches or actuators for simplified and precise motion controls without the use of complicated position- and force-control systems. To this end, we have introduced special joints to construct twisting and rotational bistable components without post-assembly. Moreover, by introducing SMPs, we demonstrated tunable bistability in both twisting and rotational components. By designing proper SMP elements, we could readjust the bistability-energy diagram after printing and achieve tunable bistable structures. This significantly increases the tunability and applicability of bistability in various 3D-printed components. Introducing SMPs into bistable components can also potentially enable stimuli-responsive motions that are useful for smart and programmable sensors and actuators^27–33^. SMPs can be used to enable active reconfiguration in bistable structures^34,35^. This could be an interesting future direction in this research.

## Methods

### Finite-element (FE) analysis

FE analysis of the 3D-printed experimental parts was performed using ABAQUS to simulate their dynamics and strain energies. A nonlinear static analysis was conducted, and the parts with little deformation and only translational/rotational motions were assumed to be rigid bodies. All materials used in the analysis were assumed to be elastic and isotropic, and the material distribution was set to be homogeneous. The temperature-dependent elastic-modulus values obtained from dynamic mechanical analysis (DMA) were used, while Poisson’s ratio was kept at 0.33. The stabilization option in ABAQUS was activated during the simulation to guarantee convergence of the solutions. Partially, full integration was used to eliminate the hourglass mode in the hexagonal meshes. More explanations are given in Supplementary Information.

### Modulus measurements

**Figure 7:**
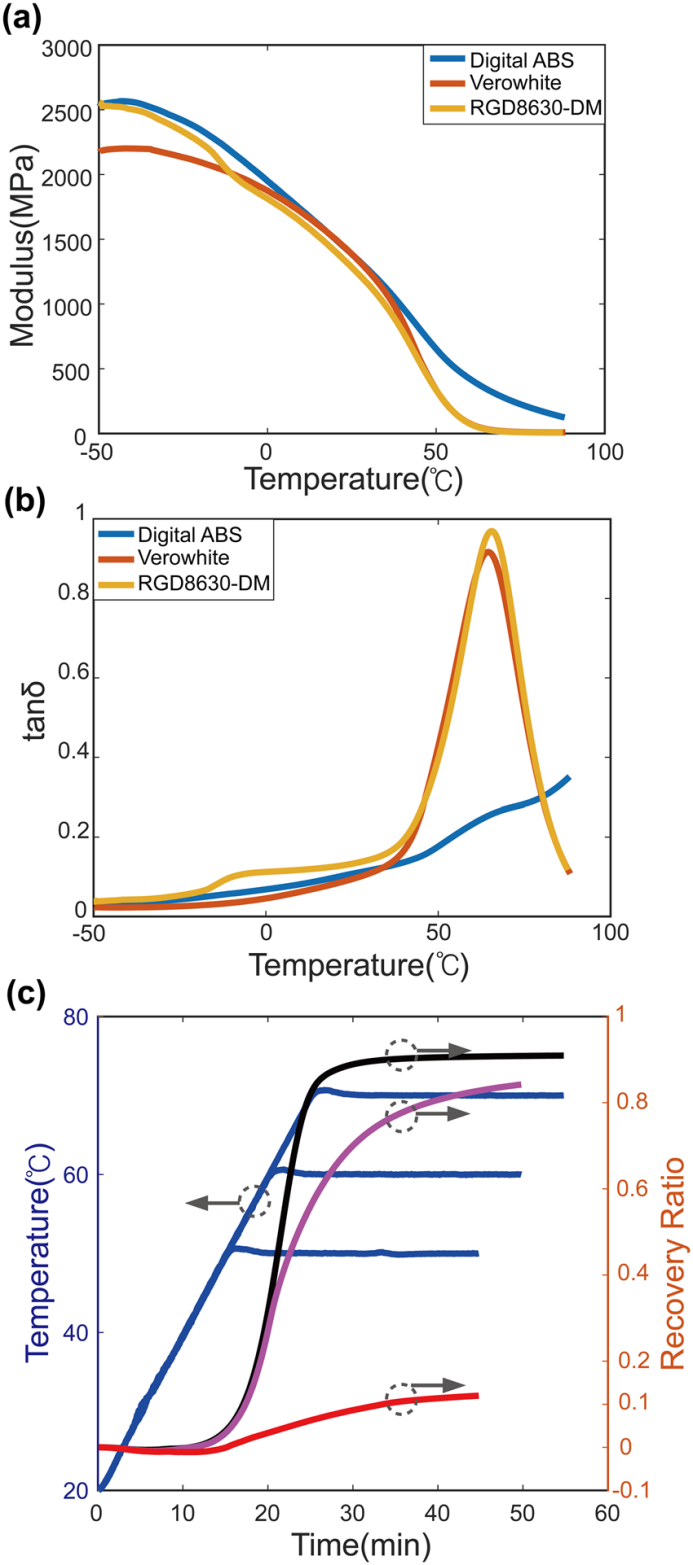
Material Characterizations. Dynamic mechanical analysis (DMA) was used to measure the temperature-dependent (**a**) modulus and (**b**) tanδ values. This figure shows data for all printing materials used in the current work. (**c**) Shows the shape-memory recovery ratio (free recovery case) for a polyJet material (RGD8530). When temperature rises up to 50 °C, 60 °C, 70 °C (blue curves), the red, pink, black curve shows the shape-memory recovery ratio at each temperature. We can note that the recovery ratio is larger for higher temperatures.

Dynamic mechanical analysis (DMA) was carried out using TA Instruments (Q800) to measure the temperature-dependent-modulus values of printing materials. Measurement samples of size 10 mm × 3 mm × 1 mm were made using the Stratasys J750 3D printer. The equilibrium was initially set at −50 °C for 5 minutes. Then, with a rate of 2 °C per minute, the samples were heated to 90 °C. During measurements, the strain oscillated with a frequency of 1 Hz at a 0.1% peak amplitude. Figure 7 shows the DMA measurement results.

## Supplementary information

Video 1

Video 2

Video 3

Supplementary Information

## Data Availability

All data generated or analysed during this study are included in this published article (and its Supplementary Information files). The datasets generated during the current study are available from the corresponding author on reasonable request.
