# Supplementary material for: 3D printing of twisting and rotational bistable structures with tuning elements
Source: Sci Rep. 2019 Jan 23;9:324. doi: 10.1038/s41598-018-36936-6 (PMC6344586; doi:10.1038/s41598-018-36936-6)
Supplement: Supplementary file 4 — Supplementary Information [file 41598_2018_36936_MOESM4_ESM.docx]

Supplementary Information for

**3D printing of twisting and rotational bistable structures with tuning elements**

Hoon Yeub Jeong^1^, Soo-Chan An^1^, In Cheol Seo^1^, Eunseo Lee^2^, Sangho Ha^2^, Namhun Kim^2^

& Young Chul Jun^1^

^1^School of Materials Science and Engineering, Ulsan National Institute of Science and Technology (UNIST), Ulsan 44919, Republic of Korea

^2^School of Mechanical, Aerospace and Nuclear Engineering, UNIST, Ulsan 44919, Republic of Korea

- Fig. S1: Mirror-symmetric images of the twisting bistable structure
- Fig. S2: Beam theory for the design of rotational bistable structures
- Fig. S3: Rotational structure with fixed-fixed beams
- Fig. S4: Twisting angle as a function of the central rod length
- Fig. S5: Design of the tuning arm in a rotational component
- Supplementary Note: Finite element simulations of bistable structures

**Supplementary Figure S1**: Mirror-symmetric images of the twisting bistable structure


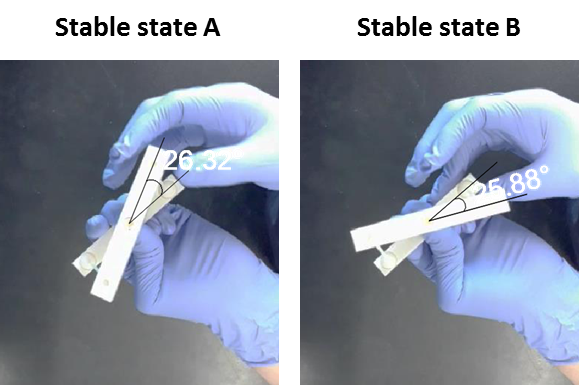


**Fig. S1.** Image capture from Supplementary Video 2. Printed ball joints can have some frictions. So, unless we push them to the end, the actual bistable mechanism may end up in a slightly different shape from the equilibrium state. In Supplementary Video 1, we pushed the mechanism only to the maximum point of the energy barrier, so that it automatically transformed to the other state. In this case, we did not obtain exact mirror-symmetric structures due to some frictions in ball joints. However, if we manually apply force further, we can obtain the exact mirror images, as shown in Fig. 2a. Supplementary Video 2 shows mirror-symmetric images more clearly. Here, we used a bigger ball joint to facilitate mechanical switching. Figure S1 shows the image capture from Video 2. A twisting bistable structure has two stable configurations, and their twisting angles are measured to be 26.32º and 25.88 º. Therefore, we obtained almost identical twisting angles with only a very small angle difference (~0.44º) and thus we have mirror images. Please note that we manually switch the mechanism, so this small angle difference can be present.

**Supplementary Figure S2:** Beam theory for the design of rotational bistable structures


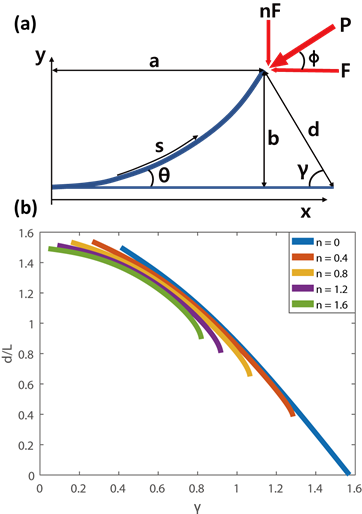


**Fig. S2.** Beam theory for the design of rotational bistable structures

Here we outline the beam theory that we need to design rotational bistable components^SR1-SR3^. a and b are defined as the x, y distances to the beam end, respectively. The notation s indicates the tangential distance along the deformed beam. θ is the angle between the beam and the x axis. θ_e_ is the angle between the end of the beam and the x axis. And

$P_{x}=Pcos\left( \phi\right)=F$ and $P_{y}=Psin\left( \phi\right)=nF$, where $n=tan(\phi)$, $P=F\sqrt{1+n^{2}}$

When the beam experiences the reverse lateral (RL) deformation, there is a strict relation between θ_e_ and n can be found:

$$2{tan}^{-1}\left( n \right)< \theta_{e}< \pi+ {tan}^{-1}(n)$$

This formula is always true in the RL deformation. The moment along the deformed beam is

$$M\left( s \right)= -nF\left( a-x\left( s \right) \right)+F(b-y\left( s \right))$$

Using $\kappa=\frac{d\theta}{ds}= \frac{M}{EI}$, the Bernoulli-Euler equation becomes

$\frac{\kappa^{2}}{2}= \frac{F}{EI}\int\left( ncos\theta-sin\theta\right)d\theta$ $=\frac{F}{EI}\left( nsin\theta+cos\theta\right)+ C_{e}$

where C_e_ is a constant in the integration. It can be found by applying the condition at the end of the beam. Given the curvature at the end of the beam is zero, we obtain

$$C_{e}= -\frac{F}{EI}(nsin\theta_{e}+cos\theta_{e})$$

Substituting it into the above equation and solving for kappa yields,

$\sqrt{2}\frac{\alpha}{L}\int_{0}^{L} ds= \int_{0}^{\theta_{e}} \frac{d\theta}{\sqrt{nsin\theta+cos\theta-nsin\theta_{e}-cos\theta_{e}}}$, where $\alpha^{2}=\frac{FL^{2}}{EI}$

Solving for alpha,

$$\alpha= \frac{1}{\sqrt{2}}\int_{0}^{\theta_{e}} \frac{d\theta}{\sqrt{nsin\theta+cos\theta-nsin\theta_{e}-cos\theta_{e}}}$$

Then,

$$\frac{a}{L}= \frac{1}{\sqrt{2}\alpha}\int_{0}^{\theta_{e}} \frac{cos\theta d\theta}{\sqrt{nsin\theta+cos\theta-nsin\theta_{e}-cos\theta_{e}}}$$

$$\frac{b}{L}= \frac{1}{\sqrt{2}\alpha}\int_{0}^{\theta_{e}} \frac{sin\theta d\theta}{\sqrt{nsin\theta+cos\theta-nsin\theta_{e}-cos\theta_{e}}}$$

These dimensionless displacement and loads are not geometry or material dependent.

$\frac{d}{L}= \sqrt{{(1-\frac{a}{L})}^{2}+ {(\frac{b}{L})}^{2}}$ and $\gamma= \frac{\pi}{2}- \tan^{-1} (\frac{1-a/L}{b/L})$

These two equations above are the stable position of the end of the beam. There is a relation between n and the position of the end of the beam in a stable configuration. In other words, once the n is determined, the specific stable position is determined in line with the n condition. Therefore, it is possible to plot the position of the end of the beam with various n, as shown in the graph below. We used this plot to design rotational bistable components.

**Supplementary Reference**

1. Howell, L. L., “Compliant Mechanisms,” John Wiley & Sons, 2001.
2. Kjell, M., “Numerical results from large deflection beam and frame problems analysed by means of elliptic integrals,” Int. J. Numer. Meth. Eng., Vol. 17, No. 1, pp. 145–153, 1981.
3. Oh, Y., “Synthesis of Multistable Equilibrium Compliant Mechanisms,” Ph.D. Thesis, University of Michigan, 2008.

**Supplementary Figure S3**: Rotational structure with fixed-fixed beams


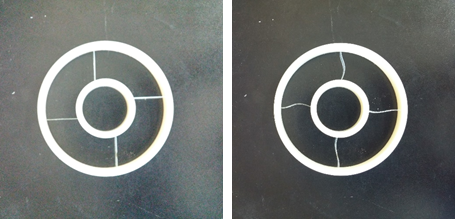


**Fig. S3.** Rotational mechanism with fixed-fixed beams. In this case, the rotational angle is much smaller (~11.4º). In Fig. 3(a), the rotation angles between two stable states were 79º and 87º for fixed-pinned beams and pinned-pinned beams, respectively.

**Supplementary Figure S4**: Twisting angle as a function of the central rod length


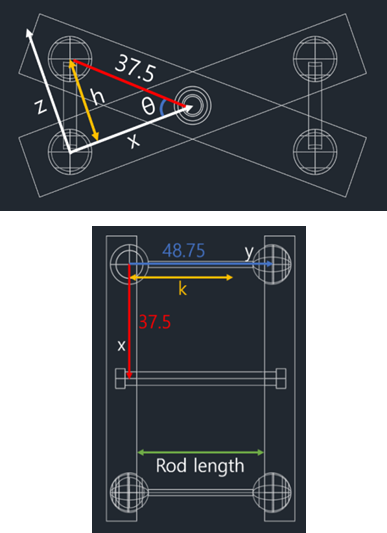


**Fig. S4**. CAD Schematic that shows how the twisting angle can be obtained as a function of the central rod length. The distance between the ball center in the joint and the rotatable central rod is 37.5 mm. The distance between two balls is 48.75mm. The direction from ball to rotatable rod will be assigned as *x* direction, while the direction between two balls *y* direction. Assuming the distance between two balls is reduced to k and the left body of the twisting structure lies in the *xy* plane, then the right body of the twisting structure rotates in the *xz* plane having a different y direction value. The beam will connect the origin point and the other ball center. The locus formula of the right ball center will be (*x*-37.5)^2^ + *z*^2^ = 37.5^2^, *y* = k. The locus formula of the beam end will be *x*^2^ + *y*^2^ + *z*^2^ = 48.75^2^.

**Supplementary Figure S5**: Design of the tuning arm in a rotational component


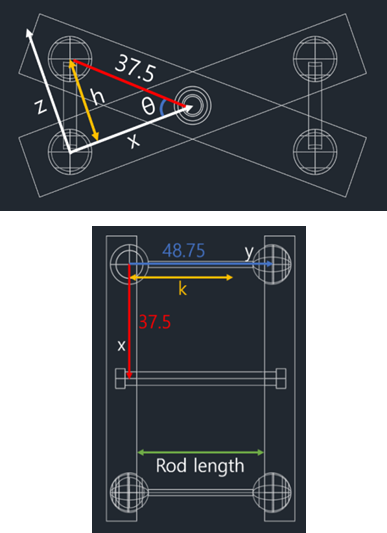


**Fig. S5**. To design a tunable rotational bistable structure, we need to solve equations to determine the required arm length in the tuning element. We first set the inner radius to be 20 mm and the outer radius 40 mm. Then we can determine the original angle to be 80° and 60° for the programmed state. In Fig. 7, 80°, 50° adjoint lines are shown. To make the 80° symmetric bistable structure, the angle between the inner arm and the line that crosses the inner arm center and pinned boundary should be 40° As a result, the required beam length is 27.83 mm. In order to make 60° bistable structure, the beam should not be on stress. Simultaneously, the inner part should be reduced and rotated. The cross-point that the 80° adjoint (red line) and unstressed beam locus (green line) meets would be a programmed arm position. The beam has a symmetric point in terms of the potential energy at the 50° adjoint line. The 80° adjoint line will make a 60° bistable structure. The red line is the symbol of the tunable arm whose length is 15.29 mm. In order to shrink and rotate this arm, we designed a structure with shrinkable half rings (made of SMPs) as shown above. This tunable arm is segmented into different parts and the end of each joining part is slanted. So, when it is plugged together by bending the SMP connecting rings, it is curved naturally. We designed the length reduction to be 2.25 mm in each segment, and then we have total 4.5mm shrinkage.

$$x\sin\theta+xsin2 \theta=15.29\cos80^{\circ}$$

$$x\cos\theta+xcos2 \theta+y=15.29\sin80^{\circ}$$

$$2x+y=15.5$$

Solving the above equations, we obtain x = 4.589 y = 6.322 and $\theta=11.27^{\circ}$. With values obtained solving equations, we determined the length of segments and the angle necessary to rotate the tuning arm.

**Supplementary Note**: Finite element simulations of bistable structures

The FE analysis of the 3D-printed experimental parts was performed using ABAQUS (nonlinear static analysis). For simplicity, joints were assumed to be frictionless, and the parts undergoing translational and rotational motion were regarded as rigid bodies. Simulations were performed using Intel Xenon CPU E5-2687W v3/3.10GHz (8 cores) and RAM 128GB. Each simulation took 20 minutes to 1 hour.

For twisting structures, a C3D10 element (10-node quadratic tetrahedron type) was used. We used structural constraints to control the degree of freedom. In the figure below, Part 2 was set as a display body (no motion). The surface of the ball joint (Part 3) was kinematically coupled to a reference point (RP1, RP3, RP4, RP6). The stabilization option in ABAQUS was used to guarantee the convergence (the damping factor was set to 0.0002). The mechanism was guided to a correct trajectory by proper boundary conditions.


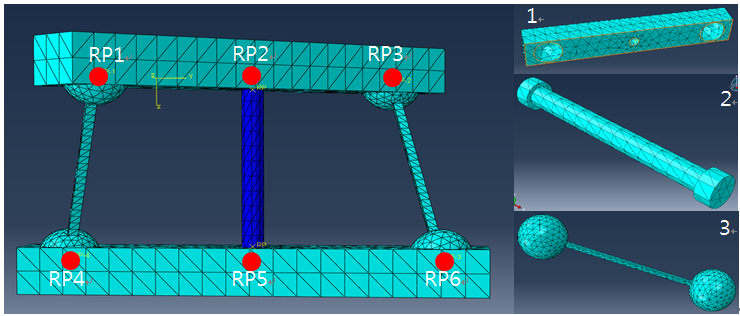


Reference points (left figure) and parts (right figure) in the twisting structure

The following pictures show the screen captures of ABAQUS simulations:


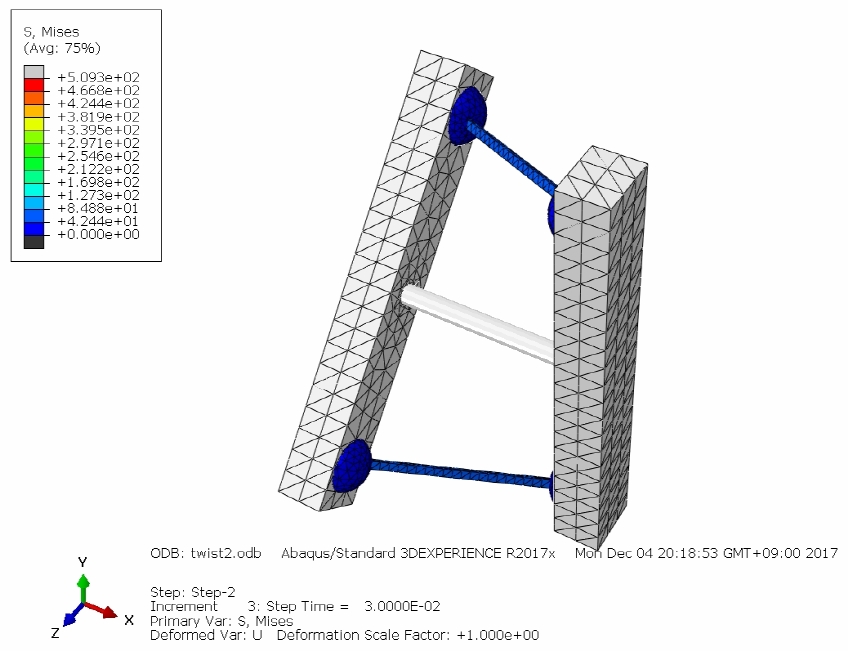


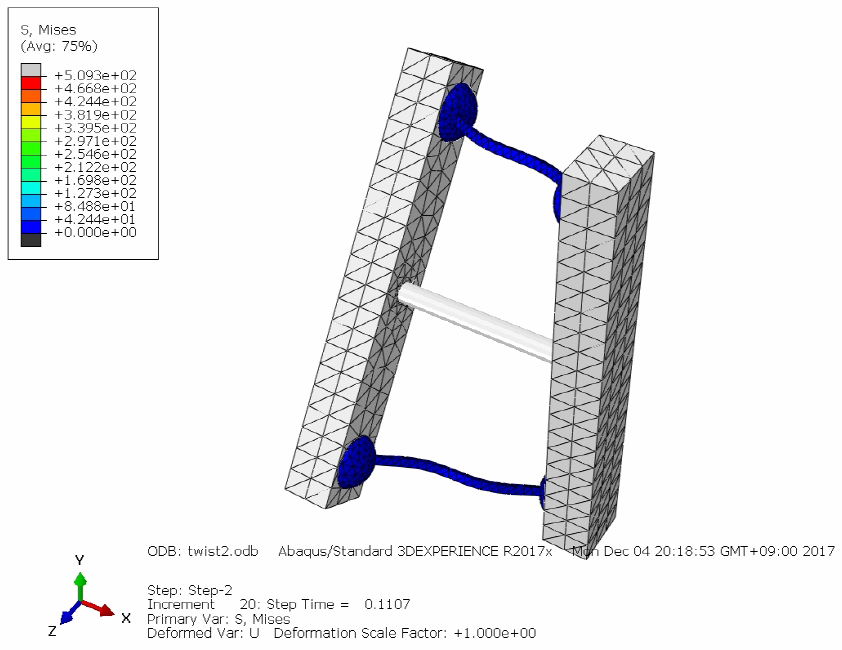


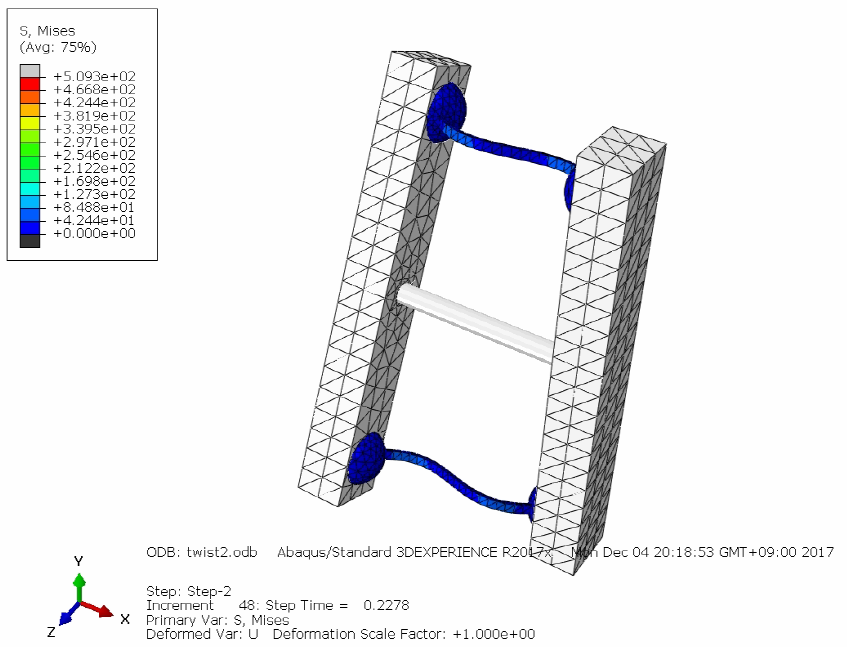


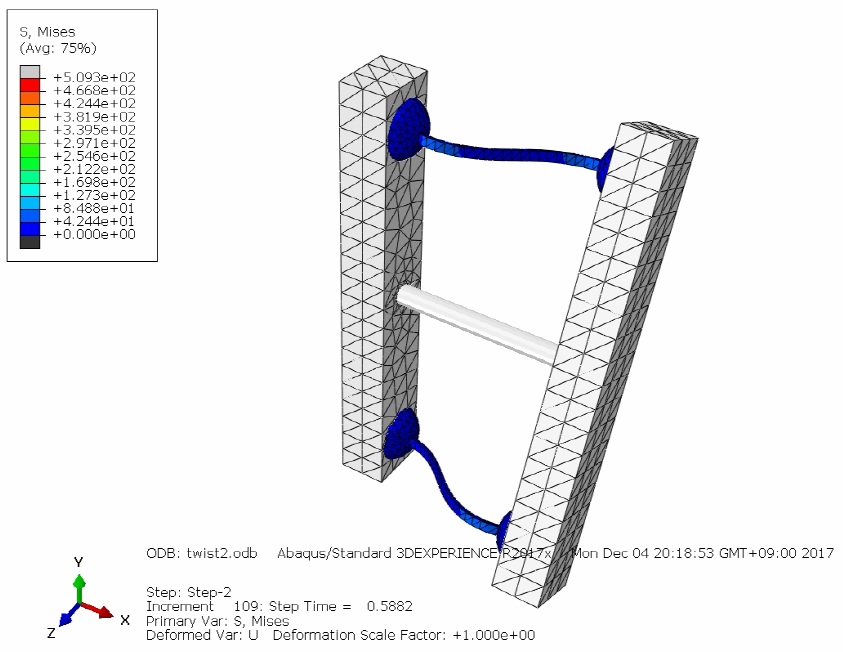


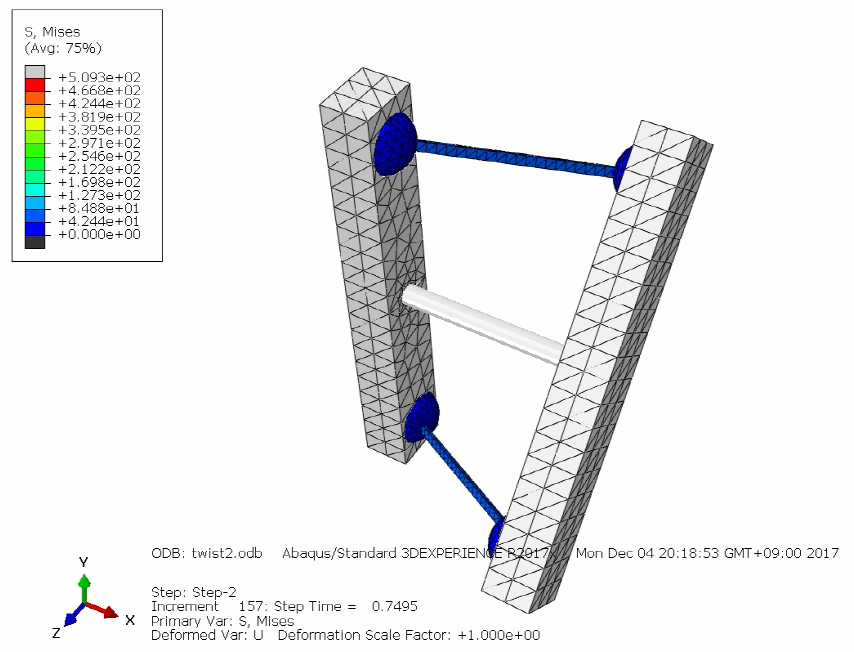


For rotational structures, C3D10 and C3D20 elements were used. In Part 1 (see the figure below), the C3D20 element was used with full integration to avoid the hourglass effect. In other parts, the C3D10 element was used (same as the twisting structure). The outer ring and Part 1 were set as rigid bodies. For the convergence of simulations, constraints were used instead of contact. RP3~RP6 has pin boundaries with Beam-type MPC constraints. The mechanism was guided to a correct trajectory by proper boundary conditions.


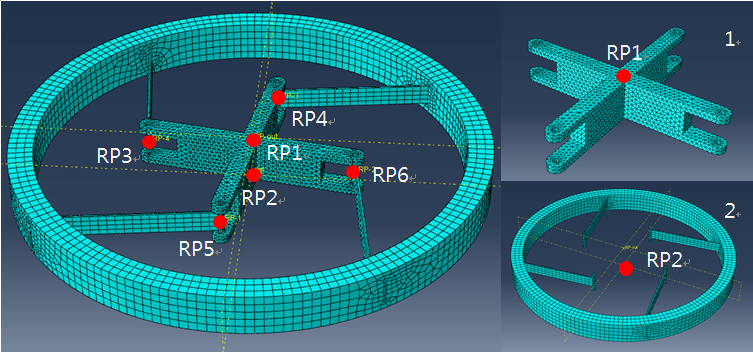


Reference points (left figure) and parts (right figure) in the rotational (pinned-fixed) structure

The following pictures show the screen captures of ABAQUS simulations:


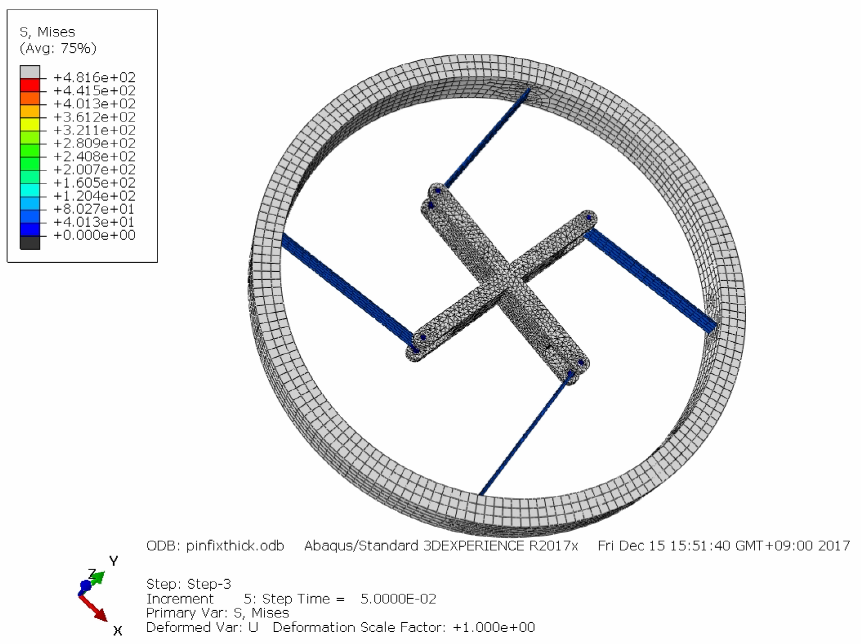


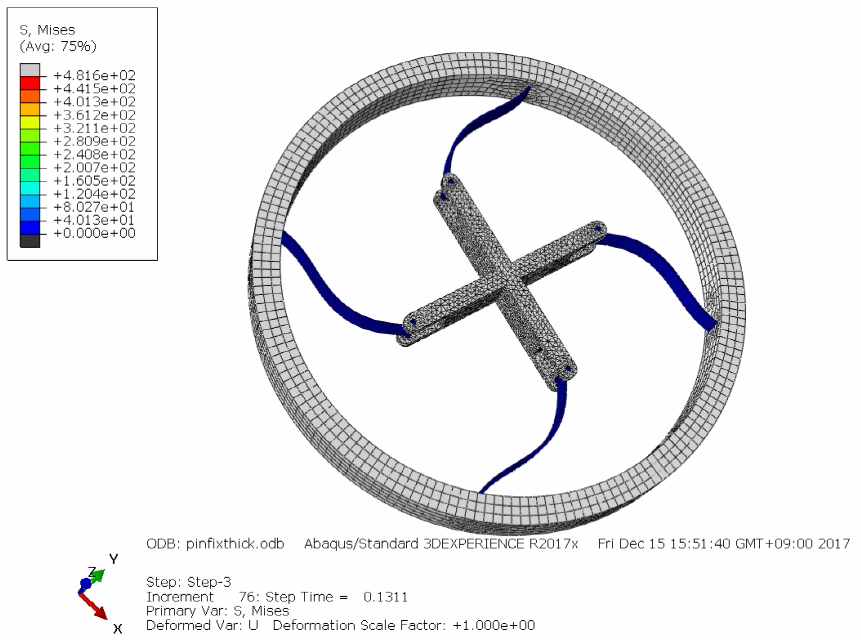


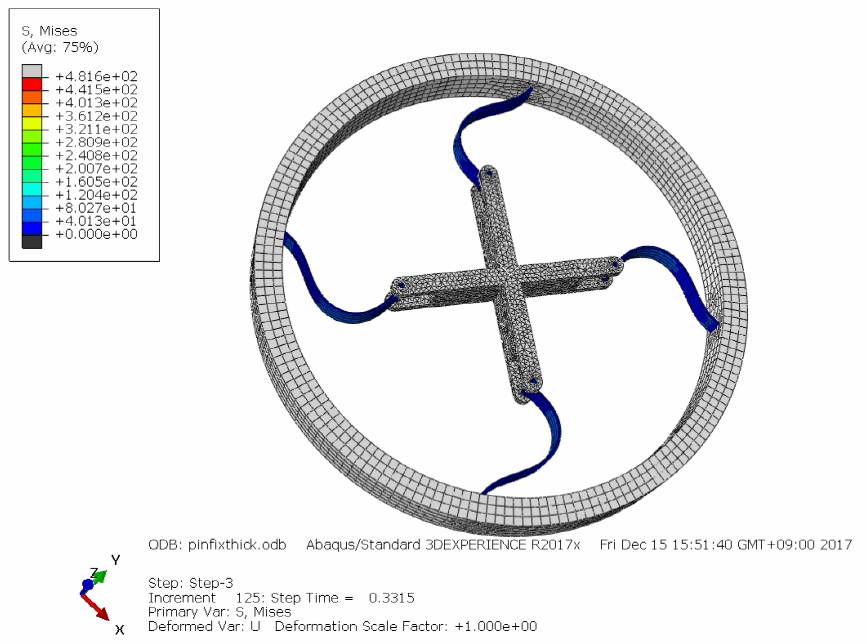


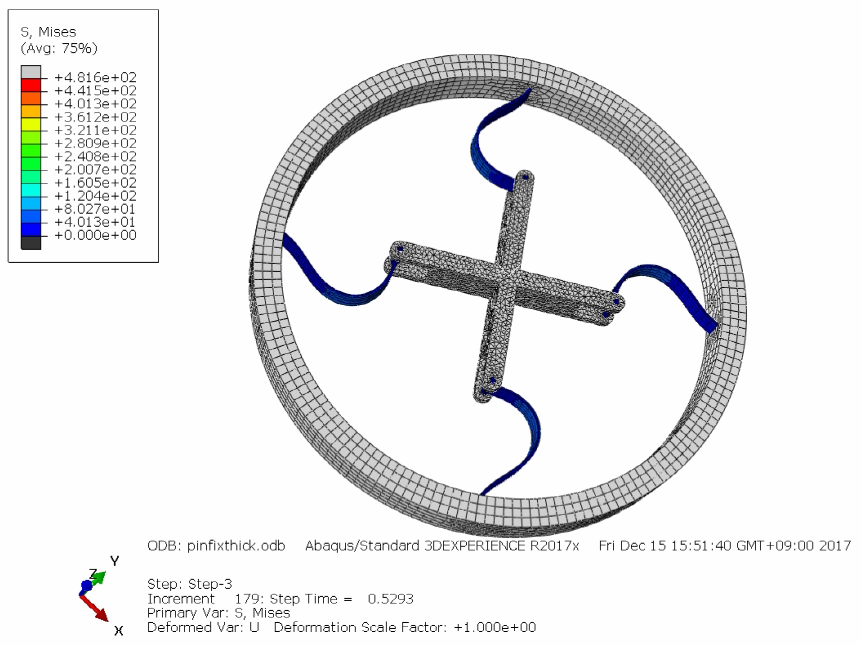


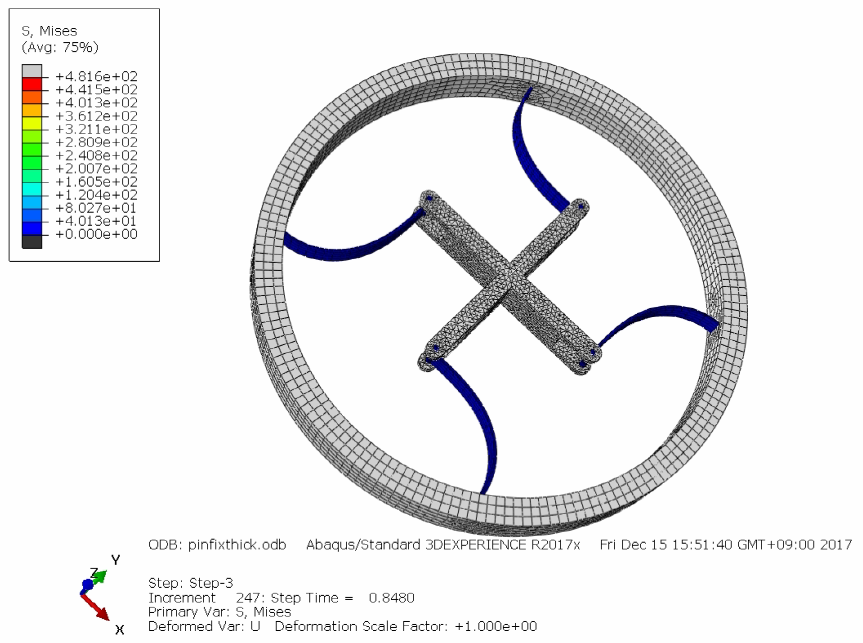


The following table compares the twisting or rotating angles from the experimental measurements of printed samples and FE simulations. We can find that they agree fairly well.

| Structure | Experiment | Simulation |
| --- | --- | --- |
| Figure 2 (Twisting) | 52.2º | 51.1º |
| Figure 3 (Rotational, pinned-fixed) | 79.3º | 79.8º |
| Figure 3 (Rotational, pinned-pinned) | 87.3º | 88.8º |
| Figure 6 (Rotational, symmetric) | 81.3º | 80.0º |
